# Predicting MicroRNA Target Genes and Identifying Hub Genes in IIA Stage Colon Cancer Patients Using Bioinformatics Analysis

**DOI:** 10.1155/2019/6341967

**Published:** 2019-02-07

**Authors:** Zhiyong Dong, Wei Lin, Stacy A. Kujawa, Shike Wu, Cunchuan Wang

**Affiliations:** ^1^Department of Gastrointestinal Surgery, The First Affiliated Hospital of Jinan University, No. 601, Huangpu Ave, Guangzhou, Guangdong, 510630, China; ^2^Department of Gastrointestinal Surgery, The Affiliated Hospital of Putian University, No. 999, Dongzhen Road, Putian, 351100, China; ^3^Robert H Lurie Comprehensive Cancer Center, Feinberg School of Medicine, Northwestern University, 303 E. Superior St., Chicago, IL, 60611, USA; ^4^Department of Gastrointestinal and Anal Surgery, Ruikang Hospital, Guangxi Traditional Chinese Medical University, No. 10, Huadong Road, Nanning 530001, China

## Abstract

**Background:**

Colon cancer is a heterogeneous disease, differing in clinical symptoms, epigenetics, and prognosis for each individual patient. Identifying the core genes is important for early diagnoses and it provides a more precise method for treating colon cancer.

**Materials and Methods:**

In this study, we wanted to pinpoint these core genes so we obtained GSE101502 microRNA profiles from the GEO database, which resulted in 17 differential expressed microRNAs that were identified by GEO2R analysis. Then, 875 upregulated and 2920 downregulated target genes were predicted by FunRich. GO and KEGG pathway were used to do enrich analysis.

**Results:**

GO analysis indicated that upregulated genes were significantly enriched in the regulation of cell communication and signaling and in nervous system development, while the downregulated genes were significantly enriched in nervous system development and regulation of transcription from the RNA polymerase II promoter. KEGG pathway analysis suggested that the upregulated genes were enriched in axon guidance, MAPK signaling pathway, and endocytosis, while the downregulated genes existed in pathways in cancer, focal adhesion, and PI3K-Akt signaling pathway. The top four molecules including 82 hub genes were identified from the PPI network and involved in endocytosis, spliceosome, TGF-beta signaling pathway, and lysosome. Finally, NUDT21, GNB1, CLINT1, and COL1A2 core gene were selected due to their correlation with the prognosis of IIA stage colon cancer.

**Conclusion:**

this study suggested that NUDT21, GNB1, CLINT1, and COL1A2 might be the core genes for colon cancer that play an important role in the development and prognosis of IIA stage colon cancer.

## 1. Introduction

Colon cancer is the second most commonly diagnosed cancer and the fourth leading cause of cancer death worldwide. It has been estimated that there were 1,360,600 new cases and 693,900 deaths of colon and rectum cancer worldwide in 2012 [1]. The American Cancer Society estimated that the incidence of colon cancer (71%) is higher than rectum cancer (29%) [2]. Colon cancer is a heterogeneous disease, differing in clinical symptoms, gene mutation or alteration, epigenetics, prognosis, and the response to therapy [3]. It is reported that multiple genes and pathways play a role in the occurrence and development of colon cancer [4]. Moreover, colon cancer is a global burden due to the rising healthcare costs to manage the disease.

MicroRNA (miRNA) is a small endogenous, noncoding RNA molecule, which is composed of approximately 21-25 nucleotides. These small miRNAs usually target one or more mRNA, regulating gene expression through translation level inhibition or breaking target mRNAs [5]. miRNAs characterize an innovative epigenetic mechanism that controls gene expression in several pathological conditions within the cancer tissues [6], and the dysfunction of miRNA is associated with different cancers. For example, Ruan et al. [7] reported that miR-1181 and miR-4314 were associated with ovarian cancer through downregulated FOXP1 and GRWD1/IP6K1/NEGR1 whereas Zhang et al. [8] indicated that the tumor suppressive role of miR-149 targeted the AKT-mTOR pathway in human hepatocellular carcinoma. miR-149-5p inhibited epithelial-to-mesenchymal transition (EMT) of cells via targeting FOXM1 in non–small cell lung cancer [9]. miR-203a-suppressed cell proliferation in human gastric cancer via targeting E2F transcription factor 3 has been described by Yang et al. [10]. Moreover, Liu et al. [11] suggested that Jun/miR-22/HuR regulatory axis may play a role in colorectal cancer progression. It is reported that no reliable biomarker profile has been identified in order to discriminate cancerous from normal tissue [12]. Many miRNA expression profiling experiments on colon cancer carcinogenesis have been published during the last several years using microarray, RNA-seq, DNA-seq, and ChIP-seq technology which have exposed hundreds of differentially expressed miRNA or genes involved in biological processes, molecular functions, or different pathways [13–15]. Therefore, how to predict genes using miRNA and identify those target genes is vital to understand the molecular mechanism, develop early diagnostics, and precisely treat colon cancer.

Gene Expression Omnibus (GEO) is an open database that provides high quantity miRNA expression data [16]. With the emerging development of high-throughput next generation sequencing in the biological sciences, the identification of core genes and the extraction of useful information from large set of gene data are essential. Therefore, we used bioinformatics analysis to solve this problem. One problem we ran into though was that miRNA targets are difficult to characterize as each miRNA has multiple gene targets so the accurate identification of miRNA and miRNA interaction remains a challenge. In this condition, several tools have been developed for miRNA target prediction with one of them being comprehensive bioinformatics analysis, which we used to analyze the expression of differential miRNA and find the core genes that exist in the development and progression of colon cancer.

In the present study, we will examine related miRNA datasets of human colon cancer from the GEO database. Overall, miRNA expression profiles of cancer tissues in patients with colon cancer were compared with those patients with normal colon tissue to identify the differential expressed miRNA. We used FunRich software to predict the target genes from the differential expressed miRNA, and the STRING (Retrieval of Interacting Genes) and Cytoscape software were used to analyze the target genes and select hub genes. Furthermore, the DVIAD online tool was used to perform enrichment analysis, and the GEPIA was used to investigate the overall survival and gene expression level of hub genes. In the end, we figured out which core genes were closely related to colon cancer, which might help researchers to examine molecular mechanisms involved in the disease prognosis, thus providing information on the precise gene therapy for colon cancer research.

## 2. Materials and Methods

### 2.1. Database and MicroRNA Selection

GEO (Gene Expression Omnibus, https://www.ncbi.nlm.nih.gov/geo/) is a public genomics database, including gene array, RNA-seq, DNA-seq, and ChIP-seq based data [16]. “Colon cancer” AND “microRNA” AND “Homo sapiens” keywords were used to search related gene expression profiles by GEO datasets. The GSE101502 profile included three IIA stage colon cancer tissues and three normal colon mucosa tissues.

### 2.2. Identifying Differentially Expressed MicroRNA

GEO2R (https://www.ncbi.nlm.nih.gov/geo/geo2r/) is an online statistics tool that allows user to compare different groups of samples to identify differential microRNA across experimental conditions. We performed a T test to identify differential microRNA. |logFC| ≥ 2 cutoff and P value < 0.05 were considered to have a statistically significant difference whereas logFC ≥ 2 was upregulated microRNA and logFC ≤ -2 was downregulated [16].

### 2.3. Predicting Target Genes

FunRich (functional enrichment) is an analysis tool used for functional enrichment and protein-protein interaction network analysis for genes or proteins. The microRNA enrichment function in FunRich could be used to perform miRNA enrichment analysis, to predict targets of microRNAs, or to find microRNAs through given target genes. Functional analysis of differentially expressed microRNA target genes was conducted to predict target genes with FunRich [17].

### 2.4. GO and KEGG Pathway Analysis of DEGs

GO (Gene Ontology) analysis is a common advantage method for annotating genes and classifying characteristic biological attributes for high-throughput genome and transcriptome data. KEGG (Kyoto Encyclopedia of Genes and Genomes) is a database used in conducting searches regarding genomes, biological pathways, diseases, drugs, and chemical substances. DAVID (Database for Annotation, Visualization and Integrated Discovery, https://david.ncifcrf.gov/) is an online bioinformatics tool that is utilized to provide the functional understanding of large lists of genes. P < 0.05 was set as the cutoff criterion. We conducted key biological processes (BP), molecular functions (MF), cellular components (CC), and pathways among those DEGs by DAVID [18, 19].

### 2.5. PPI Network and Modules Analysis

STRING (the Retrieval of Interacting Genes, https://string-db.org/) is web tool created to evaluate PPI (protein-protein interaction) networks information. To detect the potential relationship among those DEGs, we used Cytoscape software and a confidence score of ≥ 0.4 was set as the cutoff criterion. MCODE (Molecular Complex Detection) app in Cytoscape was utilized to display modules of PPI network with node score cutoff = 0.2, k-core = 2, max. depth from seed = 100, and degree cutoff = 2. Then, the top four molecules were mapped into STRING [20, 21].

### 2.6. Comparison of the Hub Genes Expression Level

The GEPIA (http://gepia.cancer-pku.cn/index.html) is an interactive online tool for analyzing the RNA-seq expression data of 9,736 tumor samples and 8,587 normal samples from the TCGA (the Cancer Genome Atlas dataset, found by NCI and NHGRI, multidimensional maps of important genomic changes in 33 types of cancer) and the GTEx projects (Genotype-Tissue Expression projects, launched by NIH, is a tissue bank and resource for biological research), with a standard processing pipeline. It offers customizable functions such as tumor and normal tissue gene differential expression analysis, and we can determine the expression of hub genes in colon cancer tissues and normal colon mucosa tissues. Survival analysis is then performed to show the high expression and low expression hub genes relationship of colon cancer and overall survival. P<0.05 was considered as significantly different. The boxplot was conducted to visualize the association between cancer and normal tissue [22].

## 3. Results

### 3.1. MicroRNA Data

The gene expression profiles for GSE101502, “microRNA expression profiling in human colon cancer”, were obtained from GEO datasets (https://www.ncbi.nlm.nih.gov/geo/). GSE101502, which was based on the GPL21439 platform (miRCURY LNA microRNA Array, 7th generation hsa, mmu, and rno [miRBase 21; probe ID version]), was submitted by Huang et al. on Jul 18th, 2017. The GSE101502 dataset contained three male patients' tissues comprised of six samples including three IIA stage colon cancer tissues and three normal colon mucosa tissues.  Table 1 showed the characteristics of tissues' information from GSE101502.

### 3.2. Identification of Differentially Expressed MicroRNA

The six samples were divided into two groups (cancer and normal tissue group), and the differentially expressed miRNA analysis was conducted by GEO2R (https://www.ncbi.nlm.nih.gov/geo/geo2r/?acc=GSE101502). P values <0.05, |LogFC > 2)| were considered as differentially expressed microRNA. LogFC > 2 was upregulated microRNA, LogFC < -2 was down-regulated.  Table 2 showed the identification of differentially expressed miRNA in the two groups.

### 3.3. Prediction of Target Genes

miRNA enrichment was used to predict potential target genes from differentially expressed miRNA. The up- and downregulated microRNA were inputted into the FunRich software tool, respectively. There were 875 up- and 2920 downregulated target genes found.

### 3.4. GO Function and KEGG Pathway Enrichment Analysis

All target genes were imported into the online analysis tool, DAVID, to identify potential GO categories and KEGG pathways. GO analysis results revealed that upregulated target genes were expressively enriched in biological processes (BP), including regulation of cell communication, regulation of signaling, and nervous system development; in molecular function (MF) including receptor signaling protein activity, transcription factor activity, RNA polymerase II core promoter proximal region sequence-specific binding, and enzyme binding; and in cell component (CC) including cell junction, cell leading edge, and adherens junction (Table 3). The downregulated target genes were expressively enriched in BP, including nervous system development, regulation of transcription from RNA polymerase II promoter, and positive regulation of RNA metabolic process; in MF including RNA polymerase II transcription factor activity, sequence-specific DNA binding, regulatory region nucleic acid binding, and regulatory region DNA binding; in CC, including nucleoplasm, neuron projection, and neuron part (Table 3). KEGG pathway analysis showed that the upregulated target genes were enriched in axon guidance, MAPK signaling pathway, endocytosis, proteoglycans in cancer, and the FoxO signaling pathway, while the downregulated target genes were enriched in pathways in cancer, focal adhesion, PI3K-Akt signaling pathway, small cell lung cancer, and signaling pathways regulating pluripotency of stem cells.  Table 4 shows the most significantly enriched pathways of the upregulated target genes and downregulated target genes were performed by KEGG analysis.

### 3.5. Module Screening and Hub Gene Selecting from the Protein-Protein Interaction (PPI) Network

**Figure 1:**
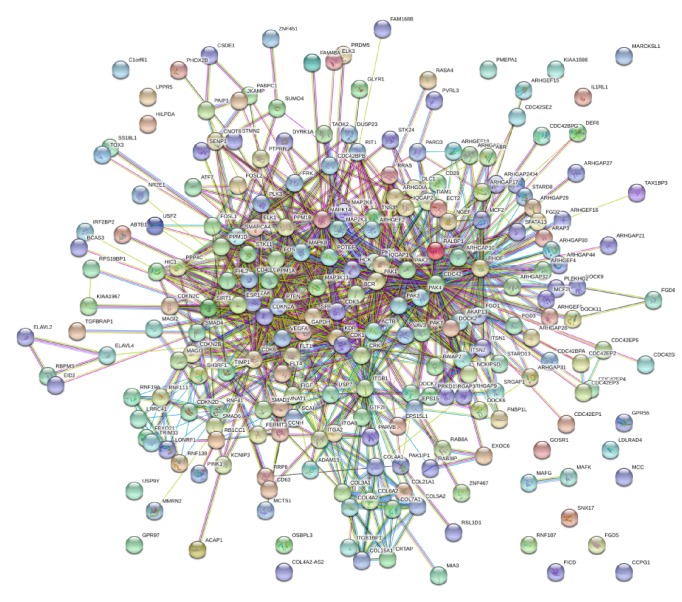
All target genes were screened and shown as association networks by STRING.

**Figure 2:**
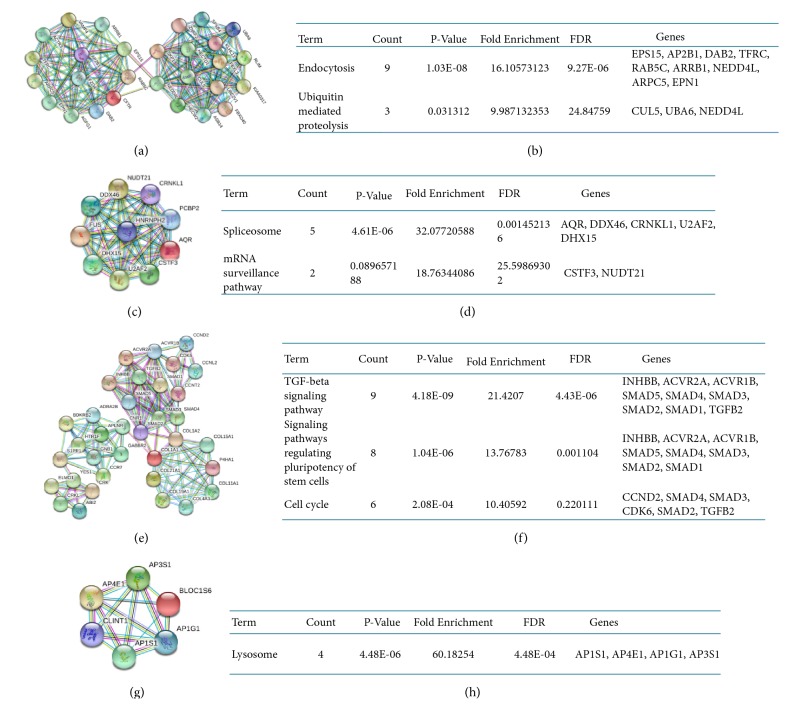
Top 4 modules from the PPI network. (a, c, e, g) modules 1 to 4; (b, d, f, h) the enriched pathway of modules 1 to 4.

All target genes were imported into the STRING database to conduct the PPI network. A combined score of > 0.4 of the nodes was considered as significance (Figure 1). Then, the results of the PPI network were exported as.txt and imported to Cytoscape software which was analyzed using plug-ins MCODE. Finally, the top four significant modules were selected and considered as hub genes. The 82 hub genes are illustrated in Figures 2(a), 2(c), 2(e), and 2(g). The functional annotations of those genes were analyzed by DAVID. Enrichment analysis indicated that the genes in modules 1 through 4 were mainly associated with endocytosis, spliceosome, TGF-beta signaling pathway, and lysosome (Figures 2(b), 2(d), 2(f), and 2(h)).

### 3.6. Survival Plots and Expression Level of Hub Genes

**Figure 3:**
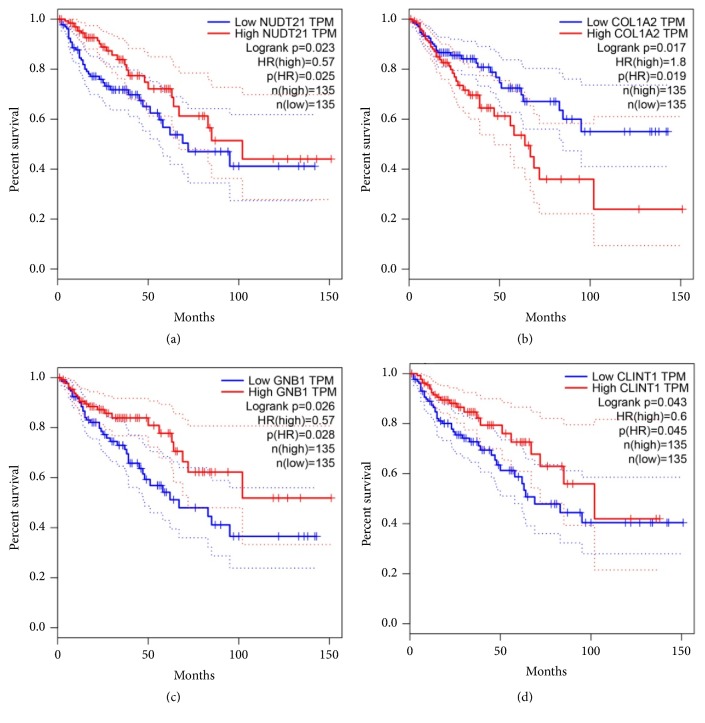
Prognostic values of the four genes (NUDT21, GNB1, CLINT1, and COL1A2) in colon cancer patients. Overall survival analysis over time with high vs. low NUDT2 expression (a), low vs. high COL1A2 expression (b), high vs. low GNB1 expression (c), and low vs. high CLINT1 expression (d). The P value was determined by log-rank test between risk groups.

**Figure 4:**
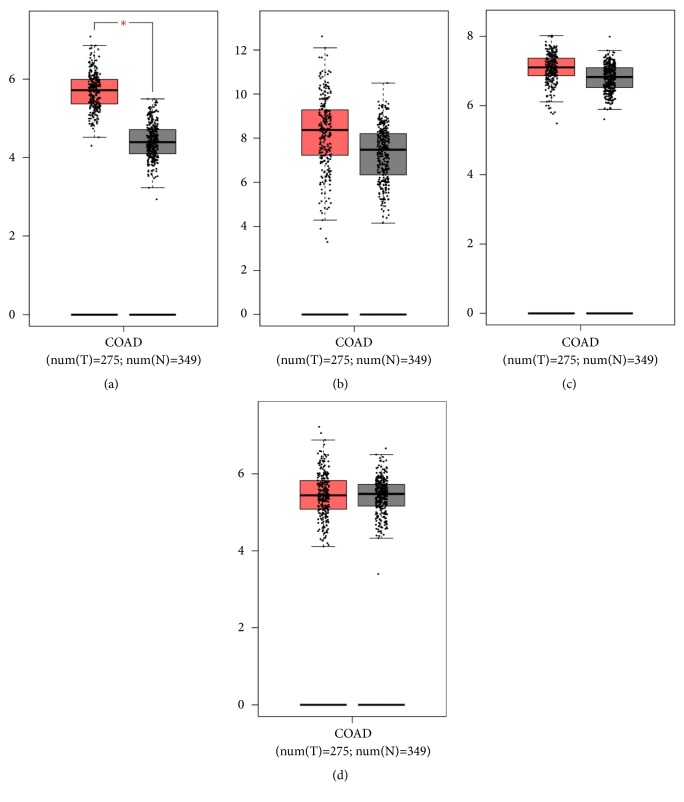
Expression levels of the four genes: NUDT21 (a), GNB1 (b), CLINT1 (c), and COL1A2 (d) in colon cancer and normal tissues. *∗*P < 0.05.

We used survival analysis by GEPIA (Gene Expression Profiling Interactive Analysis) to detect the overall survival of 82 hub genes between the high and low expression groups. It was found that high expressions of NUDT21 (HR= 0.57, P = 0.023) (Figure 1(a)), GNB1 (HR=0.028, P=0.026) (Figure 1(c)), and CLINT1 (HR=0.6, P=0.043) (Figure 1(d)) were associated with better overall survival for colon cancer patients. However, a high expression of COL1A2 (HR 1.8, P = 0.017) (Figure 1(b)) was associated with worse overall survival for colon cancer patients (Figure 3), and there was no statistical significance in the other 78 hub genes. Taken together, NUDT21, GNB1, CLINT1, and COL1A2 were considered as core genes with a close relationship to colon cancer. Then, we used GEPIA analysis to explore the core genes' expression level between colon cancer and normal tissue (Figures 4(a), 4(b), 4(c), and 4(d)).

## 4. Discussion

Pathogenesis of colon cancer is association with gene mutation, epigenetics, and the CpG island methylator phenotype [23]. In order to diagnose this disease early to precisely and effectively treat colon cancer, understanding the molecular mechanism is imperative. Microarray and high-throughput next generation sequencing have been widely utilized in order to predict the potential therapeutic targets gene of colon cancer as both techniques could provide expression levels for thousands of genes. miRNA regulates the progression of the tumor by regulating these target genes, and some miRNAs have been identified as being involved in several types of cancer [24, 25]. Therefore, it is of great significance to study the expression profile of miRNAs and predict the target genes in colon cancer. In this study, we extracted the data from GSE101502 and identified two upregulated and 15 downregulated differential expressed microRNAs between colon cancer tissue and adjacent normal mucosa tissue using bioinformatics analysis [26, 27]. And we found that NUDT21, GNB1, CLINT1, and COL1A2 might be the potential core genes that play an important role in the development and prognosis in IIA stage colon cancer.

In this study, the GO analysis showed that these potentially upregulated genes were mainly enriched in the regulation of cell communication, receptor signaling protein activity, and cell junction. Potential downregulated genes were involved in nervous system development, RNA polymerase II transcription factor activity, sequence-specific DNA binding, and nucleoplasm. Pinto et al. [28] indicated that there is a complicated cell communication in response to ionizing radiation revealed by primary human macrophage-cancer cell culture. Kim et al. [29] reported that IFITM1 expression was positively correlated with galectin-3 via receptor signaling protein activity in human colon cancer cells. The cell junctions might lead to cancer due to the differences in cell junctions for colorectal cancer [30]. Moreover, nervous system development also plays a key role in colorectal cancer metastasis [31, 32]. RNA polymerase II transcription factor contains sequence-specific DNA binding, transcriptional regulation in mammalian cells by sequence-specific DNA binding proteins [33, 34]. Between the nucleoplasm and cytoplasm called perinuclear, the signal transmission becomes abnormal by the perinucleus in malignant cell transformation [35]. All those studies indicated that the molecular functions of those up- and downregulated genes are related to colon cancer.

Moreover, the KEGG pathways for upregulated genes were enriched in axon guidance, MAPK signaling pathway, endocytosis, proteoglycans cans in cancer, and FoxO signaling pathway. Downregulated genes were involved in the pathways in cancer, focal adhesion, PI3K-Akt signaling pathway, small cell lung cancer, and regulation of signaling pathways in the pluripotency of stem cells. The axon guidance indicates that netrin 1 and Slits are causally involved in human cancer [36]. The ERK MAPK (extracellular-signal-regulated kinases) is one of the subfamilies of MAPK (mitogen-activated protein kinases), and it has been found that overexpression and activation of ERK MAPK play a role in the progression of colorectal cancer [37]. Through PIP2 mediated vinculin activation, PIPKI*γ* might positively regulate focal adhesion dynamics and colon cancer cell invasion [38]. The PI3K/AKT pathway plays an important role in the prognostic and predictive values in colorectal cancer [39]. Evidence suggests that endocytosis, proteoglycans in cancer, FoxO signaling pathway, and regulation of signaling pathways in the pluripotency of stem cells are all associated with colorectal cancer [40–42].

Finally, NUDT21, COL1A2, GNB1, and CLINT1 closely related to the overall survival of colon cancer were selected as core genes. COL1A2 is collagen type I alpha 2 chain, the fibrillary collagen detected in most connective tissues. This observation suggests that patients with a high expression of COL1A2 have a worse prognosis. Pekow et al. indicated that downregulating miR-4728-3p reduces ulcerative colitis associated colon cancers, and miR-4728-3p is a regulator of COL1A2 [43]. NUDT21 is Nudix hydrolase 21, belonging to the Nudix family of hydrolases. GNB1 is G protein subunit beta 1. Wazir et al. researched on 136 human breast cancer tissues and 31 normal tissues, undertook reverse transcription and quantitative polymerase chain reaction, and suggested that GNB1 plays an important character in the mTOR-related antiapoptosis pathway and might potentially be targeted in breast cancer [44]. CLINT1 is Clathrin interactor 1. Ajiro et al. [45] indicated that SRSF3 regulates a lot of genes including CLINT1 affecting gene expression to keep cell homeostasis. Moreover, further deeply investigated molecular mechanism of NUDT21, COL1A2, GNB1, CLINT1, and colon cancer is necessary; it is also the limitation of this study.

## 5. Conclusion

In conclusion, this study showed that NUDT21, GNB1, CLINT1, and COL1A2 might be the potential core genes that play an important role in the development and prognosis in IIA stage colon cancer. After discovering this, we have come to the conclusion that a series of experiments and further deeply investigated molecular mechanism of those four core genes should be designed to confirm the results of this study.

## Figures and Tables

**Table 1:** The characteristics of patients' tissue information in GSE101502.

**Group**	**Accession**	**Patient No.**	**Disease state**	**Stage**	**Status after 1 years**	**Age**	**Sex**	**Tissue**
Cancer group	GSM2705118	Patient 1	Primary colon cancer	TNM:IIA	alive	62	male	Cancer tissue
	GSM2705119	Patient 2	Primary colon cancer	TNM:IIA	alive	62	male	Cancer tissue
	GSM2705120	Patient 3	Primary colon cancer	TNM:IIA	alive	64	male	Cancer tissue
Normal group	GSM2705121	Patient 4	Primary colon cancer	TNM:IIA	alive	62	male	Adjacent normal mucosa
	GSM2705122	Patient 5	Primary colon cancer	TNM:IIA	alive	62	male	Adjacent normal mucosa
	GSM2705123	Patient 6	Primary colon cancer	TNM:IIA	alive	64	male	Adjacent normal mucosa

**Table 2:** Identification of differentially expressed miRNA.

	**miRNA Name**	**adj.P.Val**	**P.Value**	**t**	**B**	**logFC**
1	hsa-miR-5195-3p	0.863	0.00755	3.480604	-4.53	2.143968
2	hsa-miR-548aw	0.863	0.04462	2.481081	-4.58	2.063515
3	hsa-miR-5681a	0.863	0.02794	-2.816049	-4.57	-2.112315
4	hsa-miR-561-3p	0.863	0.04632	-2.559382	-4.58	-2.141209
5	hsa-miR-4777-3p	0.863	0.01428	-3.314158	-4.56	-2.160746
6	hsa-miR-500a-3p	0.863	0.03301	-2.541129	-4.55	-2.226463
7	hsa-miR-29c-3p	0.863	0.03073	-2.585747	-4.55	-2.232961
8	hsa-miR-200a-3p	0.863	0.03114	-2.577578	-4.55	-2.279405
9	hsa-miR-34c-3p	0.863	0.01521	-3.028324	-4.54	-2.305722
10	hsa-miR-378d	0.863	0.03592	-2.488453	-4.56	-2.354464
11	hsa-miR-142-3p	0.863	0.03736	-2.463817	-4.56	-2.488274
12	hsa-miR-4524b-3p	0.863	0.02313	-3.116108	-4.58	-2.565208
13	hsa-miR-3653-3p	0.863	0.01347	-3.105707	-4.54	-2.832038
14	hsa-miR-320c	0.863	0.00898	-3.367253	-4.54	-2.926313
15	hsa-miR-375	0.863	0.00836	-3.413574	-4.54	-3.17402
16	hsa-miR-4539	0.863	0.02251	-2.780929	-4.55	-3.278709
17	hsa-miR-215-5p	0.863	0.01006	-3.293314	-4.54	-3.955771

**Table 3:** GO analysis of differentially expressed genes associated with IIA stage colon cancer.

**regulation**	**Category**	**Term**	**Count**	%	**P-Value**	**Fold Enrichment**	**FDR**
up	GOTERM_BP_FAT	GO:0010646~regulation of cell communication	234	24.92013	2.36E-12	1.526744778	4.63E-09
up	GOTERM_BP_FAT	GO:0023051~regulation of signaling	236	25.13312	4.03E-12	1.514715803	7.91E-09
up	GOTERM_BP_FAT	GO:0007399~nervous system development	180	19.16933	8.89E-12	1.63444496	1.75E-08
up	GOTERM_BP_FAT	GO:0010604~positive regulation of macromolecule metabolic process	220	23.42918	2.72E-11	1.517826065	5.34E-08
up	GOTERM_BP_FAT	GO:0007167~enzyme linked receptor protein signaling pathway	98	10.43663	2.73E-11	2.024115217	5.37E-08
up	GOTERM_MF_FAT	GO:0005057~receptor signaling protein activity	27	2.875399	2.69E-08	3.580476019	4.39E-05
up	GOTERM_MF_FAT	GO:0000982~transcription factor activity, RNA polymerase II core promoter proximal region sequence-specific binding	41	4.366347	1.00E-06	2.33015106	0.001631
up	GOTERM_MF_FAT	GO:0019899~enzyme binding	136	14.48349	1.81E-06	1.481913682	0.002953
up	GOTERM_MF_FAT	GO:0008092~cytoskeletal protein binding	75	7.98722	3.96E-06	1.728165139	0.006455
up	GOTERM_MF_FAT	GO:0019904~protein domain specific binding	59	6.28328	4.69E-06	1.873173393	0.00764
up	GOTERM_CC_FAT	GO:0030054~cell junction	123	13.09904	2.20E-09	1.711673403	3.35E-06
up	GOTERM_CC_FAT	GO:0031252~cell leading edge	44	4.685836	2.05E-07	2.378506707	3.12E-04
up	GOTERM_CC_FAT	GO:0005912~adherens junction	69	7.348243	3.11E-07	1.907961809	4.74E-04
up	GOTERM_CC_FAT	GO:0070161~anchoring junction	69	7.348243	7.57E-07	1.862342469	0.001153
down	GOTERM_BP_FAT	GO:0007399~nervous system development	546	18.8601	3.19E-36	1.619387	6.50E-33
down	GOTERM_BP_FAT	GO:0006357~regulation of transcription from RNA polymerase II promoter	468	16.1658	4.34E-32	1.64145	8.83E-29
down	GOTERM_BP_FAT	GO:0051254~positive regulation of RNA metabolic process	375	12.95337	7.66E-29	1.705661	1.56E-25
down	GOTERM_BP_FAT	GO:0006366~transcription from RNA polymerase II promoter	453	15.64767	1.98E-28	1.601934	4.03E-25
down	GOTERM_BP_FAT	GO:1903508~positive regulation of nucleic acid-templated transcription	360	12.43523	2.90E-28	1.717546	5.91E-25
down	GOTERM_MF_FAT	GO:0000981~RNA polymerase II transcription factor activity, sequence-specific DNA binding	188	6.493955	3.54E-17	1.799249	6.12E-14
down	GOTERM_MF_FAT	GO:0001067~regulatory region nucleic acid binding	225	7.772021	7.28E-16	1.663386	1.34E-12
down	GOTERM_MF_FAT	GO:0000975~regulatory region DNA binding	224	7.737478	1.35E-15	1.657928	2.31E-12
down	GOTERM_MF_FAT	GO:0044212~transcription regulatory region DNA binding	223	7.702936	1.78E-15	1.656331	3.08E-12
down	GOTERM_MF_FAT	GO:0043565~sequence-specific DNA binding	267	9.222798	3.59E-15	1.566314	6.15E-12
down	GOTERM_CC_FAT	GO:0005654~nucleoplasm	632	21.83074	2.75E-18	1.331786	4.42E-15
down	GOTERM_CC_FAT	GO:0043005~neuron projection	233	8.048359	5.31E-12	1.522783	8.54E-09
down	GOTERM_CC_FAT	GO:0097458~neuron part	297	10.25907	8.60E-11	1.406649	1.38E-07
down	GOTERM_CC_FAT	GO:0098644~complex of collagen trimers	18	0.621762	9.04E-10	4.940876	1.45E-06

**Table 4:** KEGG pathway analysis of target genes associated with IIA stage colon cancer.

Regulated	Category	Term	Count	%	P-Value	Fold Enrichment	FDR
Up	KEGG_PATHWAY	hsa04360:Axon guidance	21	2.24	7.30E-06	3.173885	0.009462
Up	KEGG_PATHWAY	hsa04010:MAPK signaling pathway	31	3.30	1.94E-05	2.333442	0.025073
Up	KEGG_PATHWAY	hsa04144:Endocytosis	31	3.30	2.43E-05	2.306309	0.031476
Up	KEGG_PATHWAY	hsa05205:Proteoglycans in cancer	26	2.77	3.53E-05	2.495278	0.045696
Up	KEGG_PATHWAY	hsa04068:FoxO signaling pathway	20	2.13	5.61E-05	2.864842	0.072721
Down	KEGG_PATHWAY	hsa05200:Pathways in cancer	108	3.73	1.58E-10	1.810230026	2.10E-07
Down	KEGG_PATHWAY	hsa04510:Focal adhesion	66	2.28	1.44E-09	2.110470443	1.90E-06
Down	KEGG_PATHWAY	hsa04151:PI3K-Akt signaling pathway	91	3.14	4.76E-08	1.737500173	6.31E-05
Down	KEGG_PATHWAY	hsa05222:Small cell lung cancer	32	1.11	9.87E-07	2.479896821	0.001306893
Down	KEGG_PATHWAY	hsa04550:Signaling pathways regulating pluripotency of stem cells	44	1.52	2.04E-06	2.070271006	0.002694908

## Data Availability

The data used to support the findings of this study are included within the article.
